# MiR‐590‐3p regulates proliferation, migration and collagen synthesis of cardiac fibroblast by targeting ZEB1

**DOI:** 10.1111/jcmm.14704

**Published:** 2019-11-01

**Authors:** Xiaolong Yuan, Jinchun Pan, Lijuan Wen, Baoyong Gong, Jiaqi Li, Hongbin Gao, Weijiang Tan, Shi Liang, Hao Zhang, Xilong Wang

**Affiliations:** ^1^ Guangdong Provincial Key Laboratory of Laboratory Animals Guangdong Laboratory Animals Monitoring Institute Guangzhou China; ^2^ National Engineering Research Center for Swine Breeding Industry Guangdong Provincial Key Lab of Agro‐Animal Genomics and Molecular Breeding College of Animal Science South China Agricultural University Guangzhou China

**Keywords:** cardiac fibrosis, miR‐590‐3p, myocardial infarction, *ZEB1*

## Abstract

Previous studies have implicated the attractive and promising role of miR‐590‐3p to restore the cardiac function following myocardial infarction (MI). However, the molecular mechanisms for how miR‐590‐3p involves in cardiac fibrosis remain largely unexplored. Using human cardiac fibroblasts (HCFs) as the cellular model, luciferase report assay, mutation, EdU assay and transwell migration assay were applied to investigate the biological effects of miR‐590‐3p on the proliferation, differentiation, migration and collagen synthesis of cardiac fibroblasts. We found that miR‐590‐3p significantly suppressed cell proliferation and migration of HCFs. The mRNA and protein expression levels of *α‐SMA*, *Col1A1* and *Col3A* were significantly decreased by miR‐590‐3p. Moreover, miR‐590‐3p directly targeted at the 3’UTR of *ZEB1* to repress the translation of *ZEB1*. Interfering with the expression of *ZEB1* significantly decreased the cell proliferation, migration activity, mRNA and protein expressions of *α‐SMA*, *Col1A1* and *Col3A*. Furthermore, the expressions of miR‐590‐3p and *ZEB1* were identified in infarct area of MI model in pigs. Collectively, miR‐590‐3p suppresses the cell proliferation, differentiation, migration and collagen synthesis of cardiac fibroblasts by targeting *ZEB1*. These works will provide useful biological information for future studies on potential roles of miR‐590‐3p as the therapeutic target to recover cardiac function following MI.

## 1 INTRODUCTION

The acute myocardial infarction (MI) is considered to be one of the leading causes of mortality worldwide, and it has been reported that the mortality of acute MI increased by 5.6‐fold from 1987 to 2014 in China.^1^ A series of studies have reported that MI leads the death of cardiomyocytes, causing cardiac fibrosis, cardiac remodelling and heart failure.^2^, ^3^ The cardiac fibrosis following MI involves in excessive extracellular matrix (ECM) deposition^2^ and causes the changes in myocardial structure, function and phenotype.^4^ During the processes of cardiac fibrosis, MI induces and activates the collagen synthesis, proliferation and migration of fibroblasts which account for about 70% of cells in the healthy heart,^5^, ^6^ and fibroblasts migrate to the injured myocardial site and undergo differentiation to myofibroblasts to produce fibrillar collagens (types I and III)^2^, ^7^ to support the structural and shape of myocardial cells.^8^ Although a growing body of works has been carried out on cardiac fibrosis,^9^ the underlying mechanisms of cardiac fibrosis after MI remain unclear.

Currently, microRNAs (miRNAs), comprising short endogenous non‐coding RNAs of approximately 22 nucleotides in length, have been highly suggested to involve in the processes of cardiac fibrosis following MI.^10^, ^11^, ^12^ Previous studies have demonstrated that, compared to normal humans, the expression levels of miR‐26a‐1, miR‐146a and miR‐199a‐1 are significantly higher in the plasma of acute MI patients,^13^ and these miRNAs are suggested to be the potential biomarkers for acute MI diagnosis.^13^ miR‐486‐5p has been reported to suppress cardiomyocyte apoptosis and improve cardiac function though activation of PI3K/Akt pathway in rats.^14^ Compared to the wild‐type mice, double deletion of miR‐133a‐1 and miR‐133a‐2 mice show a 2.5‐fold increase in cardiomyocyte proliferation^15^ in hearts with an elevated expression of α‐smooth muscle actin (α‐SMA),^15^ which is critical for fibroblasts differentiation to myofibroblasts.^16^ miR‐185‐5p dramatically expresses lower in hearts from mice following MI than that from normal mice, and miR‐185‐5p has been confirmed to inhibit cell proliferations, migrations and tube formations at cellular level.^17^ These observations have implicated the critical functions of miRNAs in cardiac fibrosis following MI.

Previous studies have demonstrated miR‐590‐3p gets involved in cardiac fibrosis.^18^, ^19^, ^20^ The administration of synthetic miR‐590‐3p lipid formulations immediately after MI in mice results in marked reduction of infarct size and persistent recovery of cardiac function.^18^ Besides, we previously predicted that zinc finger E‐box binding homeobox 1 (ZEB1) gene, which is a transcription factor and is known for the proliferation and invasion of various cells^21^, ^22^ including human cardiomyocytes,^10^ is the potential target of miR‐590‐3p. After MI, the expression of *ZEB1* is significantly induced in mice.^23^ ZEB1 has been reported to bind at the promoter of *CXCR4* gene, which seems to restore cardiac fibrosis,^24^ to inhibit the expression of *CXCR4* in human cardiac cells to aggravate MI.^25^ Moreover, overexpression of *ZEB1* up‐regulates the expressions of collagen crosslinking enzymes as well as the expressions of *Col1A1* and *Col3A1* to mediate collagen stabilization and deposition of ECM.^26^ Therefore, we hypothesize that miR‐590‐3p gets involved in the processes of cardiac fibrosis via regulating the biological function of cardiac fibroblasts by targeting *ZEB1*.

In this study, using human cardiac fibroblasts (HCFs) as the cellular model, the biological functions of miR‐590‐3p on the proliferation, differentiation, migration and collagen synthesis of cardiac fibroblasts were first explored. The molecular regulation between miR‐590‐3p and ZEB1 were further identified, and then the biological functions of miR‐590‐3p‐mediated‐ZEB1 were characterized. These works will provide useful biological information for future studies on potential roles of miR‐590‐3p as the therapeutic target to recover cardiac function following MI.

## 2 METHODS AND MATERIALS

### 2.1 Ethics approval

All experiments in the present study were performed in accordance with the guidelines of the Animal Care and Use Committee of Guangdong Provincial Key Laboratory for Laboratory Animals and Guangdong Laboratory Animals Monitoring Institute.

### 2.2 Creation of MI model in pigs

Six young male Juema^27^ minipigs weighting 20‐25 kg were used to create MI model according to previous studies.^11^, ^28^ Briefly, these pigs were raised in Guangdong Provincial Key Laboratory for Laboratory Animals, and this laboratory has been identified and recognized by Association for Assessment and Accreditation of Laboratory Animal Care International. These anesthetized pigs were randomly divided into sham operation control group (n = 3) and MI group (n = 3). After supine bound, these pigs were transected 7‐10 cm in the left third intercostal space to expose the heart. Three MI pigs were created by permanent ligation of the trunk near one third of the apex after the first branch. The thoracic cavity was opened, and sutures were placed in the approximate position without ligation for the other three pigs for sham operation as the control group. BeneViewT5 and EDAN H100 were used to monitor the basic vital signs of animals. The success of ligation was judged and elevated by ST segment of electrocardiogram.

### 2.3 Cell culture and transfection

Human cardiac fibroblasts (HCFs) used in this study were purchased from ScienCell Research Laboratories. HCFs were cultured in Fibroblast Medium‐2 (FM‐2), incubated at 37°C in 5% CO_2_. The small interfering RNAs (siRNAs) for *ZEB1* were designed by siDirect (version 2.0, http://sidirect2.rnai.jp/) and DSIR (http://biodev.extra.cea.fr/DSIR/DSIR.html). The miR‐590‐3p mimic, inhibitor, ZEB1‐specific siRNAs and their respective negative control (NC) were synthesized and purified by RiboBio Co.Ltd.. Transfection was performed with Lipofectamine™ 3000 Reagent (Invitrogen), according to the manufacturer's protocol. Briefly, HCFs (1‐5 × 10^5^ cells/well) were seeded and cultured into six‐well plate at 1 d prior to transfection. When cells reached 70% coverage of one well, miRNAs and siRNAs were transfected into cells in antibiotic‐free medium. The transfected cells were incubated at 37°C for 4～6 hours and then replaced with the fresh complete medium. Cells were maintained in culture until other experiments.

### 2.4 Quantitative real–time polymerase chain reaction (qRT‐PCR)

The total RNA was extracted from HCFs by using Trizol reagent (Invitrogen) according to the manufacturers protocol. The quantity of RNA was assessed spectrophotometrically using a Nanodrop One (NanoDrop Technologies, Thermo). Then, 0.5 g of total RNA was reverse transcribed into cDNA using Reverse TransScript Kit (Toyobo, Takara). The mRNA expressions were performed with real‐time polymerase chain reaction (PCR) by using Maxima SYBR Green qPCR Master Mix kit (TAKARA) with *GAPDH* as the internal control in a LightCycler Real‐Time PCR system. Briefly, 20 L reactions containing 10 L of Maxima SYBR Green qPCR Master Mix, 50 ng of total RNA, 0.6 mol/L of forward primers, 0.6 mol/L of Reverse primers were subjected to one cycle of 95°C for 10 minutes and then 40 cycles of 95°C for 5s, 60°C for 60s and 72°C for 1 minutes. The relative expression of miR‐590‐3p was detected using THUNDERBIRD SYBR qPCR Kit (Toyobo, Japan) with U6 as the internal control in a LightCycler Real‐Time PCR system. Briefly, 20 L reactions containing 10 L of THUNDERBIRD SYBR qPCR Mix, 50 ng of total RNA, 0.6 mol/L of forward primers, 0.6 mol/L of Reverse primers were subjected to one cycle of 95°C for 1 minutes and then 40 cycles of 95°C for 15s, 60°C for 30s and 72°C for 1 minutes. The relative gene expression levels were calculated basing on the 2^−ΔΔ^ *^C^* ^t^ method. All procedures were repeated in at least triplicate. The primer sequences of qRT‐PCR designed by Primer Premier 5 are shown in Table 1.

**Table 1:** Primers for real‐time RT‐PCR

Name	Sequence（5′‐3′）	Product size
α‐SMA	F: GACAATGGCTCTGGGCTCTGTAA	147 bp
	R: CTGTGCTTCGTCACCCACGTA	
Col1A1	F: CCCGGGTTTCAGAGACAACTTC	148 bp
	R: TCCACATGCTTTATTCCAGCAATC	
GAPDH	F: GGATTTGGTCGTATTGGG	205 bp
	R: GGAAGATGGTGATGGGATT	
ZEB1	F: AACGCTTTTCCCATTCTGGC	167 bp
	R: TTGCCGTATCTGTGGTCGTG	
Col3A1	F: AATCAGGTAGACCCGGACGA	284 bp
	R: TCGAGCACCGTCATTACCC	
miR‐590‐3p	F: ACACTCCAGCTGGGGAGCTTATTCATAA	52 bp
	R: CAGTGCAGGGTCCGAGGTAT	
U6	F: CTGGTAGGGTGCTCGCTTCGGCAG	150 bp
	R: CAACTGGTGTCGTGGAGTCGGC	
ZEB1‐3’UTR	F: CCTCGAGTGTATGTCTTCAAACCTGGCAGT*	191 bp
	R: GCGTCGACTGTTCTACAGTCCAAGGCAAGT*	
ZEB1‐3’UTR‐MUT	MR: TCTTATCAACTTTCCA*CGCGCG*CATACTAAAATATAT*	126 bp
	MF: ATATATTTTAGTATG*CGCGCG*TGGAAAGTTGATAAGA*	
Si‐ZEB1	ATGACATGAAGCTTTGTATCTCC	23
Si‐ZEB2	CTGAAACACTGGGACATTTCATC	23
Si‐ZEB3	AGGGACTAACAATGTTAATCTGA	23

* The bold part is base protection. The italics are mutation of part of miR‐590‐3p binding site sequence.

### 2.5 Proliferation and transwell migration assay of HCFs

EdU Cell Proliferation Kit (RiboBio) was used to measure HCFs proliferation according to the manufacturer's instructions. HCFs were transfected with miR‐590‐3p mimics (25 nmol/L), miR‐590‐3p mimics control (25 nmol/L), miR‐590‐3p inhibitors (50 nmol/L), miR‐590‐3p inhibitor control (50 nmol/L) and ZEB1‐specific siRNAs (50 nmol/L) incubation 24 hours. After 24 hours of incubation, the HCFs were treated with EdU (20 µmol/L) for 2 hours at 37°C. Following fixation with 4% paraformaldehyde, permeabilized treatment with 0.5% Triton X‐100 and staining with Apollo^®^567 and Hoechst 33 342. Photographs of cells were taken using a fluorescent microscope (Nikon).

The transwell chamber (8 m pore size, Corning) was used to examine the HCFs migration ability. After transfections, the HCFs were digested with 0.25% trypsin (Hyclone) and re‐suspended with FM‐2 without FBS. The 0.6 mL of FM‐2 with 0.5% FBS was added into lower chamber. Then, 100 L of cell suspension solution was added into the upper chambers and incubated with 5% CO_2_ atmosphere at 37°C for 12 hours. After removing the medium, PBS was used to wash the migrated cells on both side of the membranes and fix the cells with 4% glutaraldehyde for 20 minutes. After removing the remained cells on the upper side of the membrane with cotton swab, the cells on the bottom side of the membranes were stained with crystal violet for 10 minutes, and the number of the cells was counted with microscope (Nikon) after washed by PBS. The cells that had migrated through the membrane were stained and counted.

### 2.6 Luciferase reporter assay

The mature sequences of miR‐590‐3p were obtained from miRbase (http://www.mirbase.org). The potential target genes for miR‐590‐3p were predicted and overlapped by three algorithms: TargetScan (http://www.targetscan.org/), miRanda (http://www.microrna.org/) and RNAhybrid (https://bibiserv.cebitec.uni-bielefeld.de/rnahybrid/). The 191 bp length of *ZEB1* 3’UTR (NCBI Accession Gene ID: 6935) containing the potential binding sites for miR‐590‐3p was cloned into pmirGLO luciferase reporter plasmid (Promega), which is designed to quantify and evaluate miRNA activity by insertion of miRNA target sites downstream of the firefly luciferase gene. Two constructs of pmirGLO luciferase reporter plasmid were generated: MUT‐ZEB1 (with mutation of part of miR‐590‐3p binding site sequence) and WT‐ZEB1 (containing the wild‐type miR‐590‐3p binding site sequence). MUT‐ZEB1 was created and built by overlapping PCR. The primers for *ZEB1* 3’UTR and MUT‐ZEB1 were listed in Table 1. The pmirGLO vector was digested with *Xho*I and *Sal*I restriction endonuclease. The DNA fragment of *ZEB1* 3’UTR contained *Xho*I and *Sal*I cleavage sites. 500 ng of the pmirGLO luciferase reporter plasmid and appropriate miRNA plasmid were co‐transfected with lipofectamine 3000 (Invitrogen) into HCFs. After transfection for 24 hours, luciferase expression was determined using the Dual‐Glo^™^ Luciferase Reporter Assay Kit (Promega) according to the manufacturer's protocol. The pRL‐TK vector (Promega) containing Renilla luciferase was also co‐transfected for normalization in all relevant experiments.

### 2.7 Western blotting

Total protein was isolated from the HCFs using the total protein kit (APPLYGEN). Then, the protein was determined using the BCA Protein Assay Kit (Thermo). The primary antibodies were α‐SMA (Absin), Col1A1 (Absin), Col13A1 (Absin) and ZEB1 (Absin) with GAPDH (Abcam) serving as an internal control. Goat anti‐rabbit IgG‐HRP was used as a secondary antibody. 80 L proteins with a pipette were electrophoresed on SDS‐PAGE and transferred onto polyvinylidene difluoride membranes (PVDF, Millipore). The membranes were blocked with 5% non‐fat milk in PBS containing a percentage of Tween‐20 for 1.5 hours and then incubated overnight at 4°C with α‐SMA (1:2000, Rabbit), Col1A1 (1:1000, Rabbit), Col13A1 (1:1000, Rabbit), ZEB1 (1:1000, Rabbit) and GAPDH (1:10 000, Rabbit) by secondary antibodies (IgG‐HRP,1:5000) for 1 hours. All proteins were visualized with the ECL‐chemiluminescent Kit and quantification was performed using densitometry with Image J software.

### 2.8 Statistical analysis

All experiments were repeated at least three times independently. All data were shown as mean ± standard deviation (SD) of repeated experiments. Student's *t* test (two‐tailed) was used to analyse the significance of mean differences in data by R software. *Indicates *P* < .05; **indicates *P* < .05; #indicates *P* > .05.

## 3 RESULTS

### 3.1 miR‐590‐3p suppresses proliferation and migration of cardiac fibroblasts

**Figure 1:**
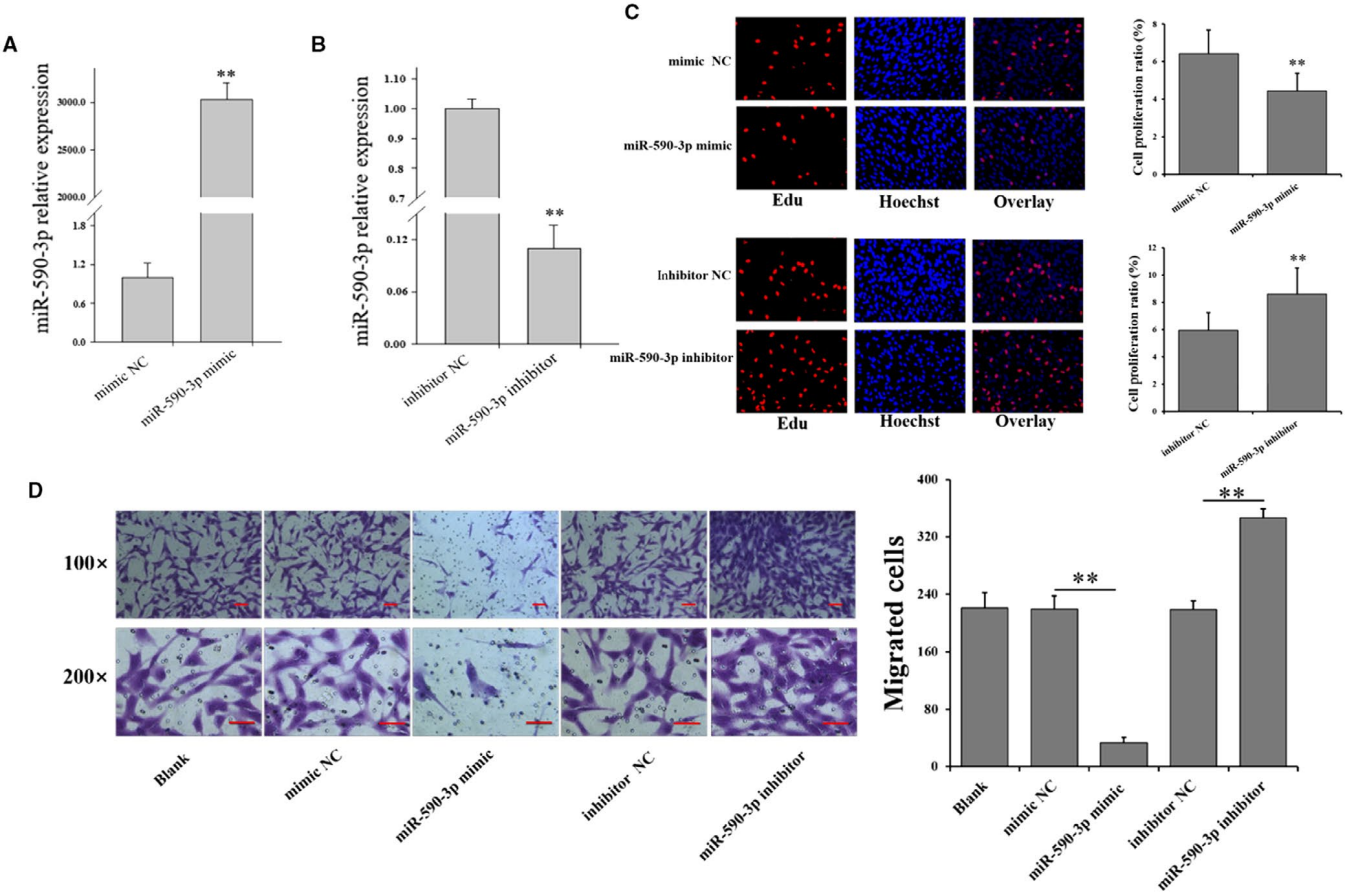
miR‐590‐3p depresses cell proliferation and migration in HCFs. miR‐590‐3p mimic (A) increased while miR‐590‐3p inhibitors (B) decreased miR‐590‐3p expression in HCFs. C, Effect of miR‐590‐3p on the proliferation of HCFs. D, Effect of miR‐590‐3p on the migration of HCFs. Bars: 100 m for 100× figures, and 50 m for 200× figures. *Indicates *P* < .05, **Indicates *P* < .01. Data are shown as mean ± SD. NC: negative control

To investigate the role of miR‐590‐3p in cardiac fibrosis, the oligonucleotide mimics or inhibitors of miR‐590‐3p were constructed and transfected into HCF cells (Figure 1). Compared to control groups, qRT‐PCR assays confirmed that miR‐590‐3p mimic significantly increased the level of miR‐590‐3p (Figure 1A), and miR‐590‐3p inhibitor significantly decreased the level of miR‐590‐3p (Figure 1B). miR‐590‐3p mimic was observed to significantly decrease the cell proliferation of HCFs (Figure 1C), and miR‐590‐3p inhibitor significantly increased the cell proliferation of HCFs (Figure 1C). Moreover, miR‐590‐3p mimic significantly decreased the migration activity of HCFs (Figure 1D, Figure S1 and S2) but miR‐590‐3p inhibitor significantly increased the migration activity of HCFs (Figure 1D, Figure S1 and S2).

### 3.2 miR‐590‐3p inhibits differentiation and collagen synthesis of cardiac fibroblasts

**Figure 2:**
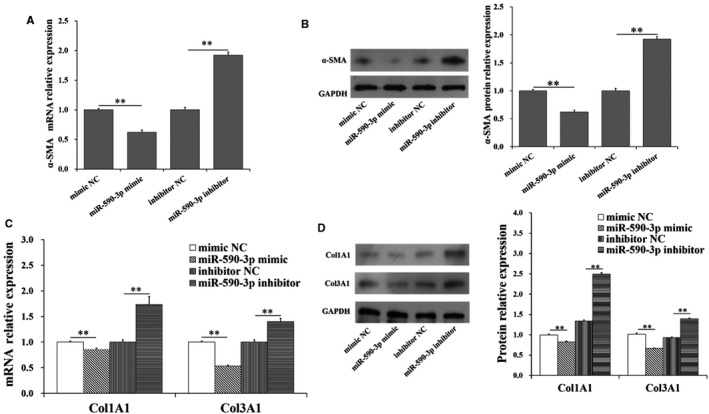
miR‐590‐3p suppresses differentiation and collagen synthesis in HCFs. The mRNA (A) and protein (B) expression of *α‐SMA* in HCFs transfected with miR‐590‐3p mimics or miR‐590‐3p inhibitor. The mRNA (C) and protein (D) expression of *Col1A1* and *Col3A1* in HCFs transfected with miR‐590‐3p mimics or miR‐590‐3p. *Indicates *P* < .05. **Indicates *P* < .01. #Indicates *P* > .05. Data are shown as mean ± SD. NC: negative control

To further explore the function of miR‐590‐3p on differentiation and collagen synthesis of HCFs, the expression levels of markers of differentiation and collagen synthesis were shown in Figure 2. Compared to control group, the mRNA and protein expression levels of *α‐SMA* were significantly decreased (Figure 2A,B) by miR‐590‐3p mimic but were significantly increased by miR‐590‐3p inhibitor (Figure 2A,B). Furthermore, miR‐590‐3p mimic was observed to significantly decrease the mRNA and protein levels of *Col1A1* and *Col3A1* (Figure 2C,D), and miR‐590‐3p inhibitor was observed to significantly increase the mRNA and protein levels of *Col1A1* and *Col3A1* (Figure 2C,D).

### 3.3 ZEB1 is a target of miR‐590‐3p

**Figure 3:**
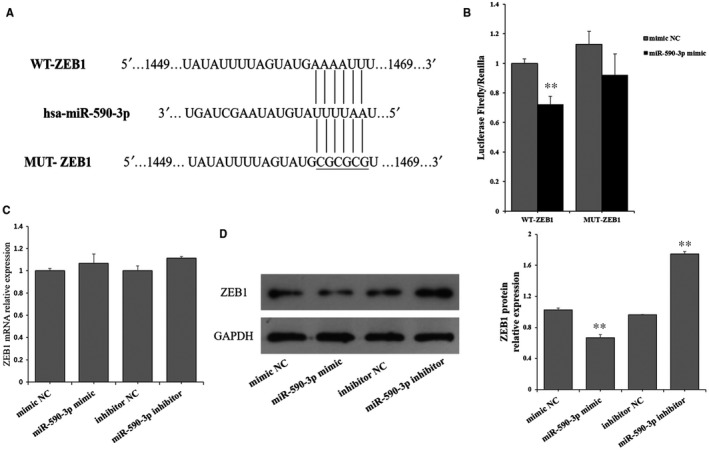
*ZEB1* is a target of miR‐590‐3p. A, Wild‐type sequence (WT‐ZEB1) and mutated sequence (MUT‐ ZEB1) for miR‐590‐3p binding site. B, Luciferase activities of WT‐ZEB1 and MUT‐ ZEB1. The mRNA (C) and protein (D) expression of ZEB1 in HCFs transfected with miR‐590‐3p mimics or miR‐590‐3p inhibitor. Data are shown as mean ± SD. *Indicates *P* < .05. **Indicates *P* < .01. NC: negative control

The putative target of miR‐590‐3p were predicted by three algorithms: TaregetScan, MiRnada and RNAhybrid. *ZEB1* gene was predicted as the overlapped potential target gene. To identify whether *ZEB1* is a target of miR‐590‐3p, the wild‐type sequence (WT‐ZEB1) and mutated sequence (MUT‐ZEB1) of the putative miR‐590‐3p binding site of ZEB1’s 3’UTR were cloned into the pmriGLO luciferase vector (Figure 3A). Compared to control group, the luciferase reporter assays showed that miR‐590‐3p mimic significantly decreased the luciferase activity of WT‐ZEB1 (Figure 3B) but did not show an obvious effect on the luciferase activity of MUT‐ZEB1 (Figure 3B). Besides, miR‐590‐3p mimic and inhibitor also did not show the significant effect on the mRNAs of *ZEB1* (Figure 3C). But miR‐590‐3p mimic significantly decreased the protein counts of ZEB1 (Figure 3D), and miR‐590‐3p inhibitor significantly increased the protein counts of ZEB1 (Figure 3D).

### 3.4 miR‐590‐3p suppresses the proliferation and migration by targeting ZEB1

**Figure 4:**
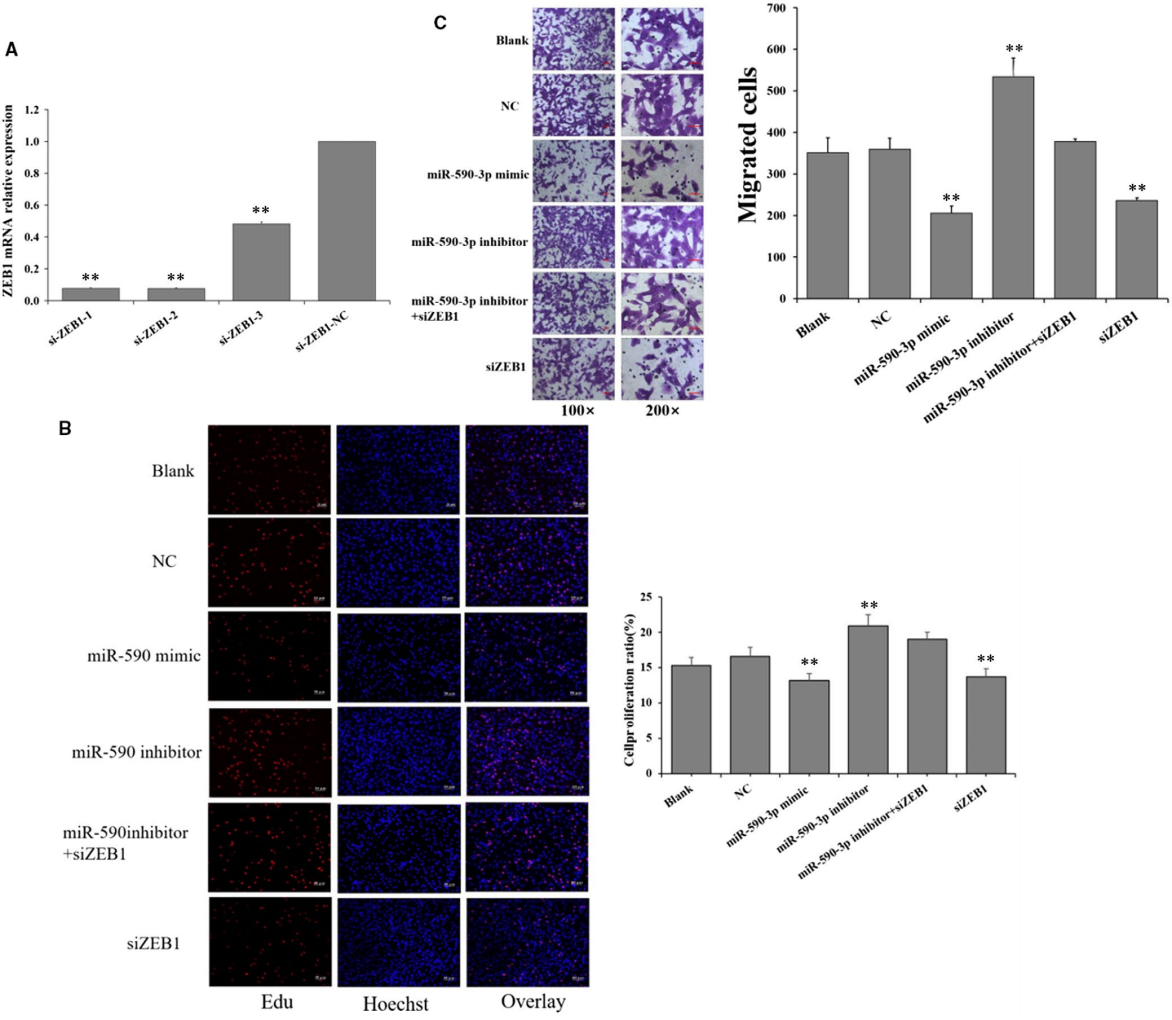
miR‐590‐3p suppresses the proliferation, migration, and collagen synthesis by targeting *ZEB1*. A, The relative expression of *ZEB1* was knockdown by three siRNAs. Effect of interfering with the expression of *ZEB1* on the cell proliferation (B) and migration (C) in HCFs. Data are shown as mean ± SD. *Indicates *P* < .05. **Indicates *P* < .01. Bars: 100 m for 100× figures, and 50 m for 200× figures. NC: negative control. SiZEB1: small interfering RNA for *ZEB1* gene

**Figure 5:**
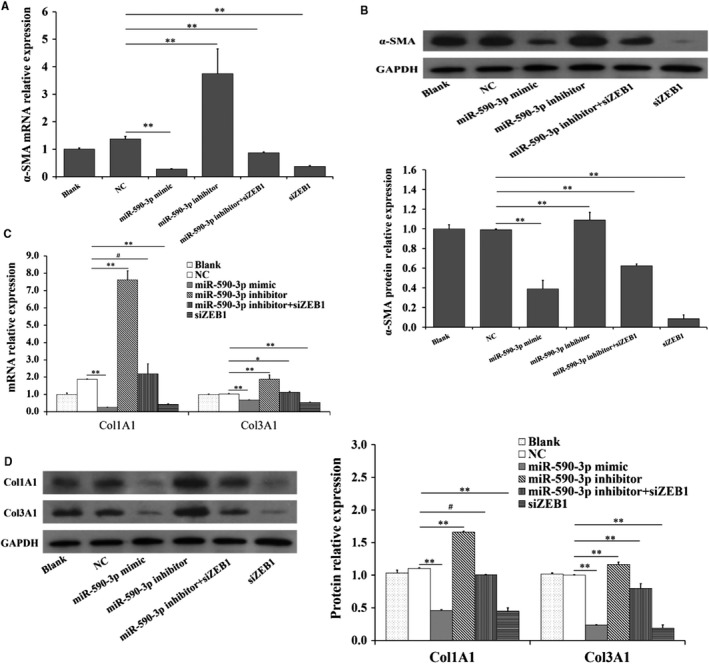
miR‐590‐3p suppresses the differentiation and collagen synthesis by targeting *ZEB1*. Effect of interfering with the expression of *ZEB1* on the mRNAs (A) and protein (B) levels of *α‐SMA.* Effect of interfering with the expression of *ZEB1* on the mRNAs (C) and protein (D) levels of *Col1A1* and *Col3A1*. Data are shown as mean ± SD. *Indicates *P* < .05. **Indicates *P* < .01. NC: negative control. SiZEB1: small interfering RNA for *ZEB1* gene

To further explore whether miR‐590‐3p inhibits the proliferation and migration of cardiac fibroblast by targeting *ZEB1* in HCFs, the expressions of *ZEB1* was interfered with specific siRNAs (Figures 4 and 5). Three ZEB1‐specific siRNAs (si‐ZEB1‐1, si‐ZEB1‐2, and si‐ZEB1‐3) and a negative control (si‐ZEB1‐NC) were transfected into HCFs. As shown in Figure 4A, si‐ZEB1‐1 exhibited the highest inhibition efficiency and thus was selected for knockdown of *ZEB1* in HCFs. Compared to NC, siZEB1 significantly decreased the cell proliferation (Figure 4B) and migration activity (Figure 4C) of HCFs, and this observation was in line with that of miR‐590‐mimic (Figure 4B,C). Compared to NC, miR‐590‐3p inhibitor significantly increased the cell proliferation (Figure 4B) and migration activity (Figure 4C) of HCFs, but miR‐590‐3p inhibitor + siZEB1 could reverse the biological functions of miR‐590‐3p inhibitor on the proliferation and migration of cardiac fibroblast (Figure 4B,C).

### 3.5 miR‐590‐3p inhibits the differentiation and collagen synthesis by targeting ZEB1

Compared to NC group, siZEB1 was observed to significantly decrease the mRNA (Figure 5A,C) and protein (Figure 5B,D) expressions of *α‐SMA, Col1A1* and *Col3A1*, and this observation was according to that of miR‐590‐3p mimic (Figure 5). Moreover, miR‐590‐3p inhibitor significantly increased the mRNA (Figure 5A,C) and protein (Figure 5B,D) expressions of *α‐SMA, Col1A1* and *Col3A1*, but the miR‐590‐3p inhibitor + siZEB1 could reverse the biological effects of miR‐590‐3p inhibitor (Figure 5).

### 3.6 Expression of miR‐590‐3p and *ZEB1* in infarct area of MI model based on miniature pigs

**Figure 6:**
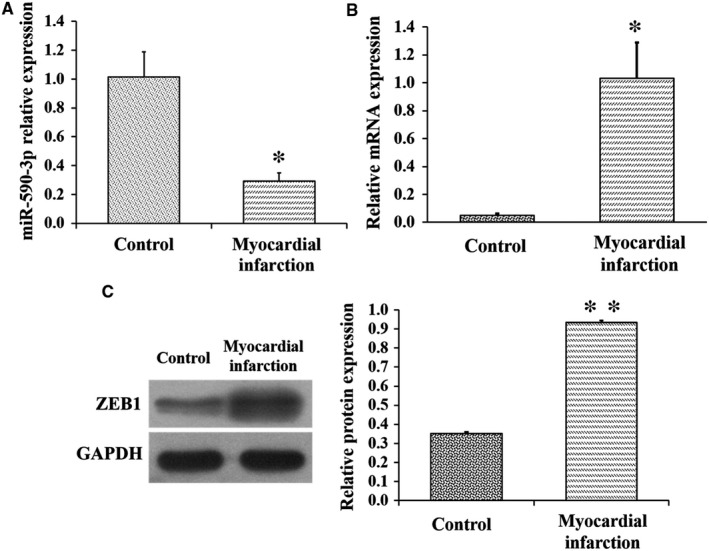
Expression of miR‐590‐3p and *ZEB1* in MI model of pigs. A, Expression of miR‐590‐3p at infarct area in control and MI pigs. The mRNA (B) and protein (C) expression levels of *ZEB1* at infarct area in control and MI pigs. Data are shown as mean ± SD. *Indicates *P* < .05. **Indicates *P* < .01

To further explore the biological functions of miR‐590‐3p and *ZEB1* in cardiac fibroblasts after MI, the MI model was created and built in minipigs. Compared to control pigs, the expression of miR‐590‐3p in infarct area was significantly lower in MI pigs (Figure 6A), but the mRNA (Figure 6B) and protein (Figure 6C) expression levels of *ZEB1* in infarct area were significantly higher in MI pigs. These observations were in line with the supposed role of miR‐590‐3p and *ZEB1* in cardiac fibroblast.

## 4 DISCUSSION

Previous studies have shown that MI causes cardiac fibrosis, cardiac remodelling and heart failure^2^, ^3^ due to inadequate regeneration of cardiomyocytes. The cardiac fibroblasts produce excessive extracellular matrix deposition^2^ to support myocardial structure^4^ and regulate cardiomyocyte proliferation and growth by directly connecting to cardiomyocytes via connexins.^29^ Although previous studies have suggested the potential roles of miR‐590‐3p as the therapeutic target to recover cardiac function following MI, the biological role of miR‐590‐3p in cardiac fibrosis following MI remain vitally unexplored. In the present study, miR‐590‐3p was observed to regulate the proliferation, differentiation, migration and collagen synthesis of cardiac fibroblasts. As shown in Figures 1 and 2, miR‐590‐3p was observed to significantly decrease cell proliferation (Figure 1C) and migration activity of HCFs (Figure1D). Moreover, miR‐590‐3p was found to significantly decrease the mRNA and protein expression levels of *α‐SMA* (Figure 2A,B), which was considered to be the marker for fibroblasts differentiation to myofibroblasts.^16^ Also, miR‐590‐3p was observed to significantly decrease the mRNA and protein levels of *Col1A1* and *Col3A1* (Figure 2C,D). These results suggest that miR‐590‐3p suppresses the proliferation, differentiation, migration and collagen synthesis of cardiac fibroblasts.

In cancer cells, miR‐590‐3p has been often reported to suppress the cell proliferation and migration.^30^, ^31^, ^32^ For example, in head and neck cancer cells, miR‐590‐3p is reported to suppress the expression of *Cyclin B* and *Cdk1*, which are key regulators of cell cycle progression, and then reduce cell proliferation and migration.^31^ In hepatocellular carcinoma cells, miR‐590‐3p suppresses cell growth in vitro and in vivo by targeting *TEAD1*.^33^ In intrahepatic cholangiocarcinoma cells, miR‐590‐3p dramatically suppresses cell migration and invasion.^32^ In breast cancer cells, miR‐590‐3p suppresses cell survival and triggers cell apoptosis via targeting sirtuin‐1 and deacetylation of p53.^30^ In breast cancer cells, miR‐590‐3p has been suggested to decrease cell proliferation and increase cell apoptosis.^34^ These findings coupling with the observations in this study support that miR‐590‐3p suppresses the proliferation, differentiation, migration and collagen synthesis of cardiac fibroblasts.

Moreover, miR‐590‐3p has been shown to be able to attenuate the cardio sphere‐derived cells differentiation, which is one type of stem cells in cardiac cell lineages.^35^ The administration of synthetic miR‐590‐3p lipid formulations immediately after MI in mice results in marked reduction of infarct size and persistent recovery of cardiac function.^18^ After MI in mice, miR‐590‐3p is reported to stimulate marked cardiac regeneration and almost complete recovery of cardiac functional parameters.^36^ These findings recommend the attractive and promising role of miR‐590‐3p to restore the cardiac function. These observations suggest the possibility that miR‐590‐3p suppresses the proliferation, differentiation, migration and collagen synthesis of cardiac fibroblasts and thus promotes the cardiomyocyte proliferation following MI.

In this study, *ZEB1* was predicted as the potential target of miR‐590‐3p by three algorithms: TaregetScan, MiRnada, and RNAhybrid and miR‐590‐3p mimic significantly decreased the luciferase activity of WT‐ZEB1 (Figure 3B) but did not show an obvious effect on the luciferase activity of MUT‐ZEB1 (Figure 3B). Although miR‐590‐3p did not show a significant effect on the mRNAs of *ZEB1*, miR‐590‐3p significantly decreased the protein counts of *ZEB1* (Figure 3C,D). Moreover, compared to NC group, siZEB1 was observed to reverse the biological functions of miR‐590‐3p inhibitor on the cell proliferation, migration, mRNA and protein expressions of *α‐SMA Col1A1* and *Col3A1* in cardiac fibroblasts (Figures 4A,B and 5). These results recommend that miR‐590‐3p suppresses the proliferation, differentiation, migration and collagen synthesis of cardiac fibroblasts by targeting *ZEB1*.

*ZEB1* gene is a transcription factor and has been reported to promote cell proliferation and invasion in carcinoma cells,^37^, ^38^ osteosarcoma cells 21, 22 and glioblastoma cells.^39^ *ZEB1* has been demonstrated to regulate the expression of genes encoding inflammatory cytokines related to poor prognosis in patients with breast cancer.^40^ Inhibition of *ZEB1* facilitates osteosarcoma cell apoptosis and inhibits cell proliferation and invasion.^41^ These observations are in line with this study that cell proliferation and migration activity of HCFs are significantly inhibited by interfering with the expression of *ZEB1* (Figure 4A,B), and these findings are according with that miR‐590‐3p suppress cell proliferation and migration of cardiac fibroblasts (Figure 1C,D) and cancer cells.^30^, ^31^, ^32^ Furthermore, *ZEB1* significantly induces the mRNA expression of *Col1A1* and *Col3A1* of ECM‐related genes to dive lung cancer invasion and metastasis,^26^ and this report is according to our study that the mRNA and protein expressions of *Col1A1* and *Col3A1* are significantly decreased by interfering with the expression of *ZEB1* (Figure 5C,D). These results demonstrate that *ZEB1* promotes the proliferation, differentiation, migration and collagen synthesis of cardiac fibroblasts. Collectively, it is possible that miR‐590‐3p suppresses proliferation, differentiation, migration and collagen synthesis of cardiac fibroblasts by targeting *ZEB1* and thus regulates the proliferation and differentiation cardiomyocytes during the processes of cardiac fibrosis following MI.

## CONFLICT OF INTEREST

The authors declare no competing interests.

## AUTHOR CONTRIBUTIONS

XLY, HZ, and XLW conceived and designed this work; XLY, JCP, BYG and HBG acquired the biological samples and analysed the data; LJW, JCP and XLY conducted the experiment; LJW and XLY drafted the work; HZ, XLW, JQL, WJT and SL revised the draft critically; All authors reviewed and approved the final manuscript.

## Data Availability

The datasets used in the current study are available from the corresponding author on reasonable request.

## References

[1] Chang J , Liu X , Sun Y . Mortality due to acute myocardial infarction in China from 1987 to 2014: secular trends and age‐period‐cohort effects. Int J Cardiol. 2017;227:229‐238. 27839815 10.1016/j.ijcard.2016.11.130

[2] Shinde AV , Frangogiannis NG . Fibroblasts in myocardial infarction: a role in inflammation and repair. J Mol Cell Cardiol. 2014;70:74‐82. 24321195 10.1016/j.yjmcc.2013.11.015 PMC3995820

[3] Lajiness JD , Conway SJ . Origin, development, and differentiation of cardiac fibroblasts. J Mol Cell Cardiol. 2014;70:2‐8. 24231799 10.1016/j.yjmcc.2013.11.003 PMC3995835

[4] Kurose H , Mangmool S . Myofibroblasts and inflammatory cells as players of cardiac fibrosis. Arch Pharm Res. 2016;39(8):1100‐1113. 27515051 10.1007/s12272-016-0809-6

[5] Zak R . Development and proliferative capacity of cardiac muscle cells. Circ Res, 1974;35(2):17‐26. 4276486

[6] Eghbali M , Blumenfeld OO , Seifter S , et al. Localization of types I, III and IV collagen mRNAs in rat heart cells by in situ hybridization. J Mol Cell Cardiol. 1989;21(1):103‐113. 10.1016/0022-2828(89)91498-3 2716064

[7] Chistiakov DA , Orekhov AN , Bobryshev YV . The role of cardiac fibroblasts in post‐myocardial heart tissue repair. Exp Mol Pathol. 2016;101(2):231‐240. 27619160 10.1016/j.yexmp.2016.09.002

[8] Covell JW . Factors influencing diastolic function. Possible role of the extracellular matrix. Circulation. 1990;81(2 Suppl):p. III155‐8. 2297881

[9] Felisbino MB , McKinsey TA . Epigenetics in cardiac fibrosis: emphasis on inflammation and fibroblast activation. JACC Basic Transl Sci. 2018;3(5):704‐715. 30456341 10.1016/j.jacbts.2018.05.003 PMC6234501

[10] Shi K , Sun H , Zhang H , Xie D , Yu B . miR‐34a‐5p aggravates hypoxia‐induced apoptosis by targeting ZEB1 in cardiomyocytes. Biol Chem. 2019;400(2):227‐236. 30312158 10.1515/hsz-2018-0195

[11] Gabisonia K , Prosdocimo G , Aquaro GD , et al. MicroRNA therapy stimulates uncontrolled cardiac repair after myocardial infarction in pigs. Nature. 2019;569(7756):418‐422. 31068698 10.1038/s41586-019-1191-6 PMC6768803

[12] Wang GK , Zhu JQ , Zhang JT , et al. Circulating microRNA: a novel potential biomarker for early diagnosis of acute myocardial infarction in humans. Eur Heart J. 2010;31(6):659‐666. 20159880 10.1093/eurheartj/ehq013

[13] Xue S , Zhu W , Liu D , et al. Circulating miR‐26a‐1, miR‐146a and miR‐199a‐1 are potential candidate biomarkers for acute myocardial infarction. Mol Med. 2019;25(1):18. 31092195 10.1186/s10020-019-0086-1 PMC6521554

[14] Zhu HH , Wang XT , Sun YH , et al. MicroRNA‐486‐5p targeting PTEN protects against coronary microembolization‐induced cardiomyocyte apoptosis in rats by activating the PI3K/AKT pathway. Eur J Pharmacol. 2019;855:244‐251. 31075240 10.1016/j.ejphar.2019.03.045

[15] Liu N , Bezprozvannaya S , Williams AH . microRNA‐133a regulates cardiomyocyte proliferation and suppresses smooth muscle gene expression in the heart. Genes Dev. 2008;22(23):3242‐3254. 19015276 10.1101/gad.1738708 PMC2600761

[16] Willems IE , Havenith MG , De Mey JG , Daemen MJ . The alpha‐smooth muscle actin‐positive cells in healing human myocardial scars. Am J Pathol. 1994;145(4):868‐875. 7943177 PMC1887334

[17] Li CC , Qiu XT , Sun Q , et al. Endogenous reduction of miR‐185 accelerates cardiac function recovery in mice following myocardial infarction via targeting of cathepsin K. J Cell Mol Med. 2019;23(2):1164‐1173. 30450725 10.1111/jcmm.14016 PMC6349160

[18] Lesizza P , Prosdocimo G , Martinelli V , Sinagra G , Zacchigna S , Giacca M . Single‐dose Intracardiac injection of pro‐regenerative MicroRNAs improves cardiac function after myocardial infarction. Circ Res. 2017;120(8):1298‐1304. 28077443 10.1161/CIRCRESAHA.116.309589

[19] Lin X , Steinberg S , Kandasamy SK et al. Common miR‐590 variant rs6971711 present only in African Americans reduces miR‐590 biogenesis. PLoS ONE. 2016;11(5):e0156065. 27196440 10.1371/journal.pone.0156065 PMC4873136

[20] He Q , Wang F , Honda T , James J , Li J , Redington A . Loss of miR‐144 signaling interrupts extracellular matrix remodeling after myocardial infarction leading to worsened cardiac function. Sci Rep. 2018;8(1):16886. 30443020 10.1038/s41598-018-35314-6 PMC6237773

[21] Yan H , Zhang B , Fang C , Chen L . miR‐340 alleviates chemoresistance of osteosarcoma cells by targeting ZEB1. Anticancer Drugs. 2018;29(5):440‐448. 29494357 10.1097/CAD.0000000000000614

[22] Han XX , Liu F , Zhang C , Ren Z , Li L , Wang G . SIAH1/ZEB1/IL‐6 axis is involved in doxorubicin (Dox) resistance of osteosarcoma cells. Biol Chem. 2019;400(4):545‐553. 30265649 10.1515/hsz-2018-0292

[23] Li J , Cai SX , He Q , et al. Intravenous miR‐144 reduces left ventricular remodeling after myocardial infarction. Basic Res Cardiol. 2018;113(5):36. 30084039 10.1007/s00395-018-0694-x

[24] Das S , Goldstone AB , Wang H , et al. A unique collateral artery development program promotes neonatal heart regeneration. Cell. 2019;176(5):1128‐1142.e18. 30686582 10.1016/j.cell.2018.12.023 PMC6435282

[25] Beji S , Milano G , Scopece A , et al. Doxorubicin upregulates CXCR4 via miR‐200c/ZEB1‐dependent mechanism in human cardiac mesenchymal progenitor cells. Cell Death Dis. 2017;8(8):e3020. 28837147 10.1038/cddis.2017.409 PMC5596590

[26] Peng DH , Ungewiss C , Tong P , et al. ZEB1 induces LOXL2‐mediated collagen stabilization and deposition in the extracellular matrix to drive lung cancer invasion and metastasis. Oncogene. 2017;36(14):1925‐1938. 27694892 10.1038/onc.2016.358 PMC5378666

[27] Chen GS , Chen S , Sui YA . Effect of slaughter weight on production and meat quality of Juema pig. Indian Journal of Animal Research. 2016;50(4):588‐594.

[28] Lopez E , Sánchez‐Margallo FM , Álvarez V , et al. Identification of very early inflammatory markers in a porcine myocardial infarction model. BMC Vet Res. 2019;15(1):91. 30898123 10.1186/s12917-019-1837-5 PMC6427889

[29] Lajiness JD , Conway SJ . The dynamic role of cardiac fibroblasts in development and disease. J Cardiovasc Transl Res. 2012;5(6):739‐748. 22878976 10.1007/s12265-012-9394-3 PMC3740345

[30] Abdolvahabi Z , Nourbakhsh M , Hosseinkhani S , et al. MicroRNA‐590‐3P suppresses cell survival and triggers breast cancer cell apoptosis via targeting sirtuin‐1 and deacetylation of p53. J Cell Biochem. 2019;120(6):9356‐9368. 30520099 10.1002/jcb.28211

[31] Di Agostino S , Valenti F , Sacconi A , et al. Long non‐coding MIR205HG Depletes Hsa‐miR‐590‐3p leading to unrestrained proliferation in head and neck squamous cell carcinoma. Theranostics. 2018;8(7):1850‐1868. 29556360 10.7150/thno.22167 PMC5858504

[32] Zu C , Liu S , Cao W , et al. MiR‐590‐3p suppresses epithelial‐mesenchymal transition in intrahepatic cholangiocarcinoma by inhibiting SIP1 expression. Oncotarget. 2017;8(21):34698‐34708. 28423728 10.18632/oncotarget.16150 PMC5471004

[33] Ge X , Gong LS . MiR‐590‐3p suppresses hepatocellular carcinoma growth by targeting TEAD1. Tumor Biology. 2017;39(3). 10.1177/1010428317695947 28349829

[34] Rohini M , Gokulnath M , Miranda PJ , Selvamurugan N . miR‐590‐3p inhibits proliferation and promotes apoptosis by targeting activating transcription factor 3 in human breast cancer cells. Biochimie. 2018;154:10‐18. 30076901 10.1016/j.biochi.2018.07.023

[35] Ekhteraei‐Tousi S , Mohammad‐Soltani B , Sadeghizadeh M , Mowla SJ , Parsi S , Soleimani M . Inhibitory effect of Hsa‐miR‐590‐5p on cardiosphere‐derived stem cells differentiation through downregulation of TGFB signaling. J Cell Biochem. 2015;116(1):179‐191. 25163461 10.1002/jcb.24957

[36] Eulalio A , Mano M , Ferro MD , et al. Functional screening identifies miRNAs inducing cardiac regeneration. Nature. 2012;492(7429):376‐381. 23222520 10.1038/nature11739

[37] Roy BC , Kohno T , Iwakawa R , et al. Involvement of LKB1 in epithelial‐mesenchymal transition (EMT) of human lung cancer cells. Lung Cancer. 2010;70(2):136‐145. 20207041 10.1016/j.lungcan.2010.02.004

[38] Zhang PJ , Sun YT , Ma L . ZEB1: At the crossroads of epithelial‐mesenchymal transition, metastasis and therapy resistance. Cell Cycle. 2015;14(4):481‐487. 25607528 10.1080/15384101.2015.1006048 PMC4614883

[39] Siebzehnrubl FA , Silver DJ , Tugertimur B , et al. The ZEB1 pathway links glioblastoma initiation, invasion and chemoresistance. EMBO Mol Med. 2013;5(8):1196‐1212. 23818228 10.1002/emmm.201302827 PMC3944461

[40] Katsura A , Tamura Y , Hokari S , et al. ZEB1‐regulated inflammatory phenotype in breast cancer cells. Mol Oncol. 2017;11(9):1241‐1262. 28618162 10.1002/1878-0261.12098 PMC5579340

[41] Xu XM , Liu W , Cao ZH , Liu MX . Effects of ZEB1 on regulating osteosarcoma cells via NF‐kappaB/iNOS. Eur Rev Med Pharmacol Sci. 2017;21(6):1184‐1190. 28387915

