# Supplementary material for: MiR‐590‐3p regulates proliferation, migration and collagen synthesis of cardiac fibroblast by targeting ZEB1
Source: J Cell Mol Med. 2019 Nov 1;24(1):227–37. doi: 10.1111/jcmm.14704 (PMC6933374; doi:10.1111/jcmm.14704)
Supplement: Supplementary file 1 [file JCMM-24-227-s001.docx]

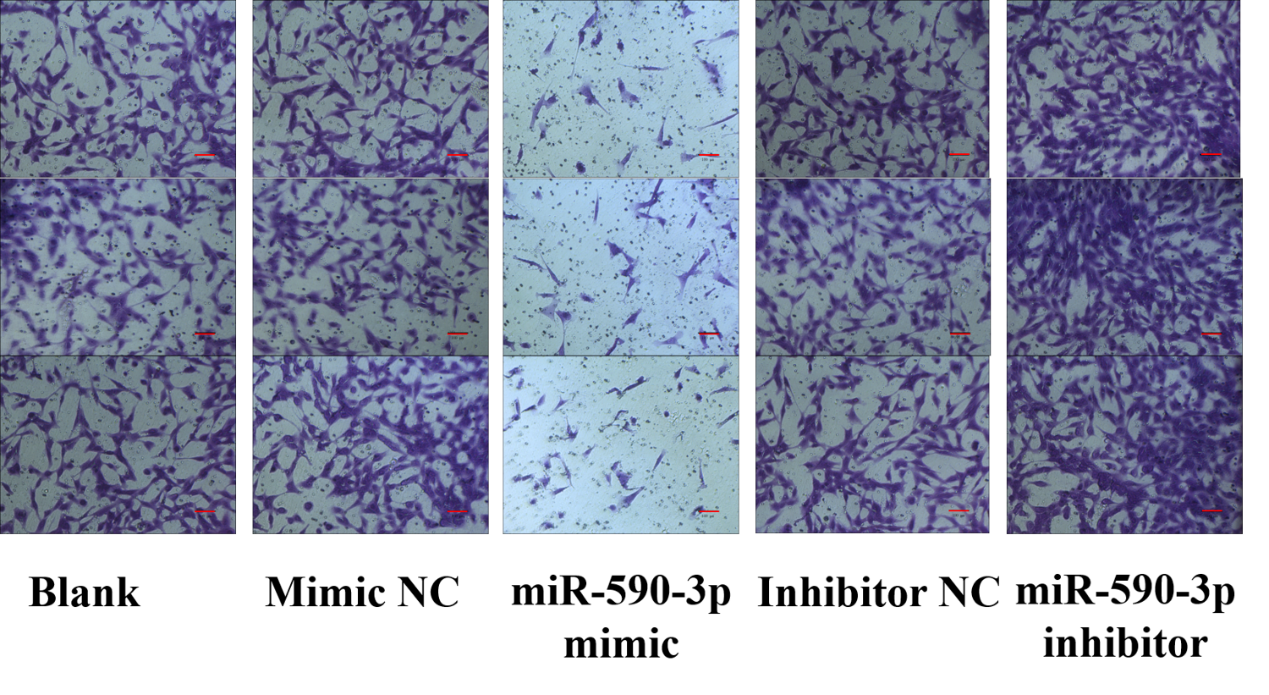


**Figure S1. Effect of miR-590-3p on the migration of Human Cardiac Fibroblasts (100**$\boldsymbol{\times}$**)**. Bars: 100 μm. NC: negative control.


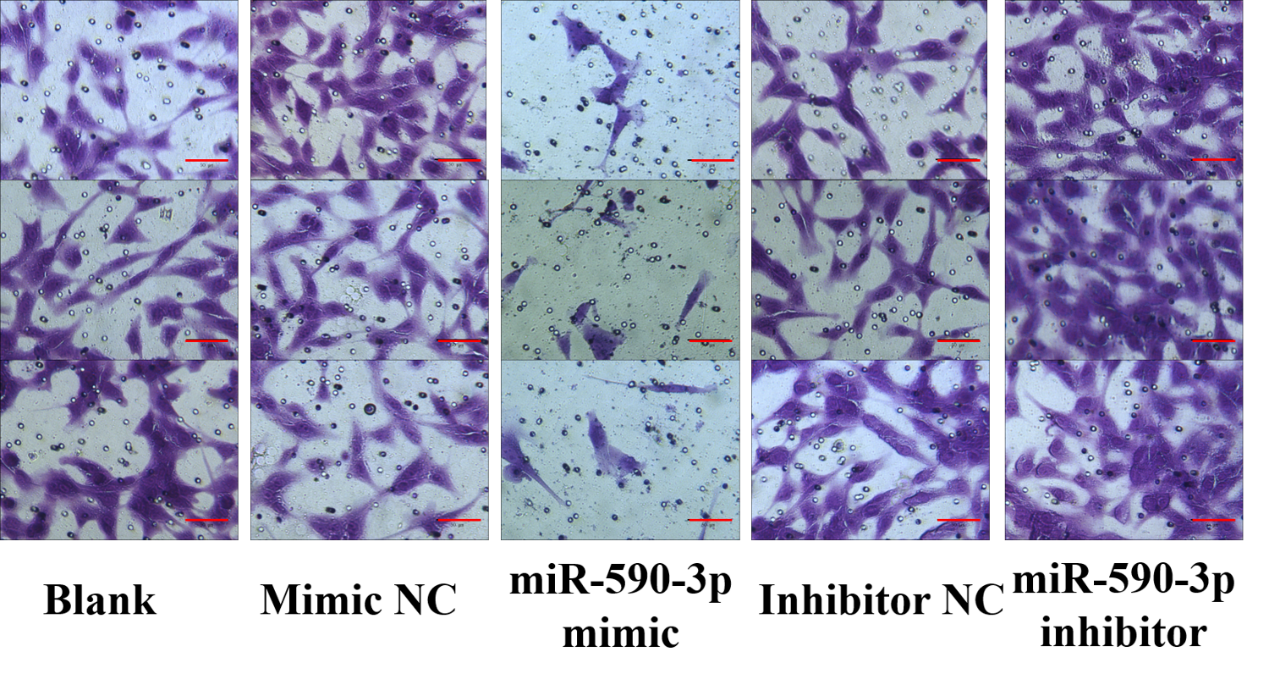


**Figure S2. Effect of miR-590-3p on migration of Human Cardiac Fibroblasts (200**$\boldsymbol{\times}$**)**. Bars: 50 μm. NC: negative control.


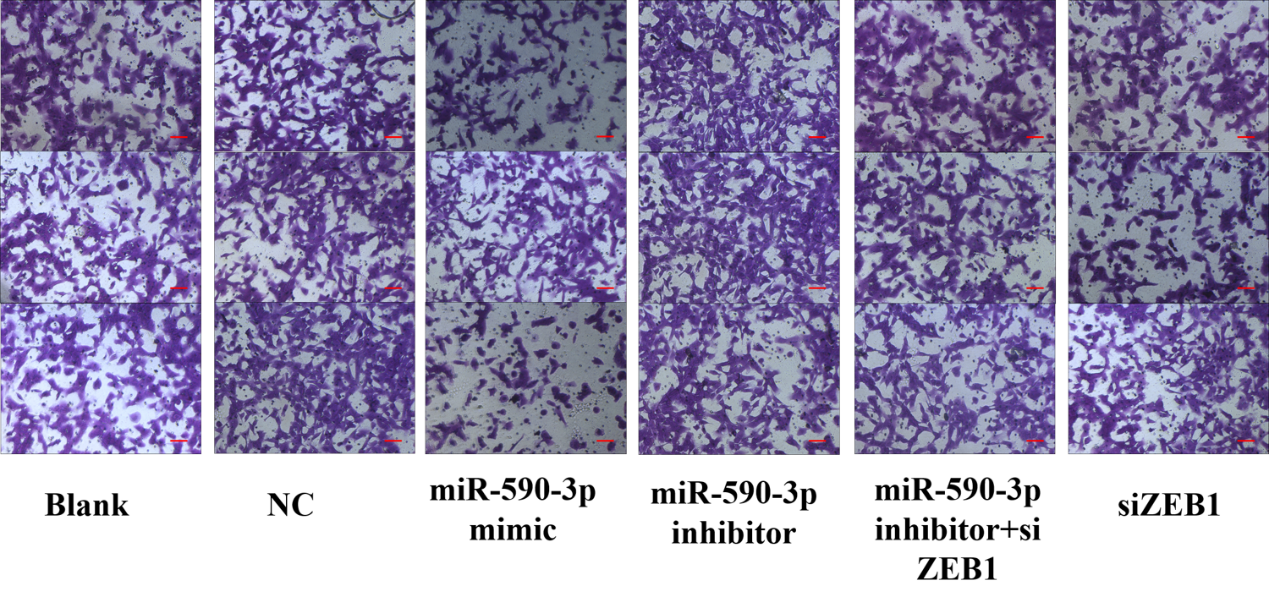


**Figure S3. Effect of interfering with the expression of ZEB1 on migration of Human Cardiac Fibroblasts (100**$\boldsymbol{\times}$**)**. Bars: 100 μm. NC: negative control.


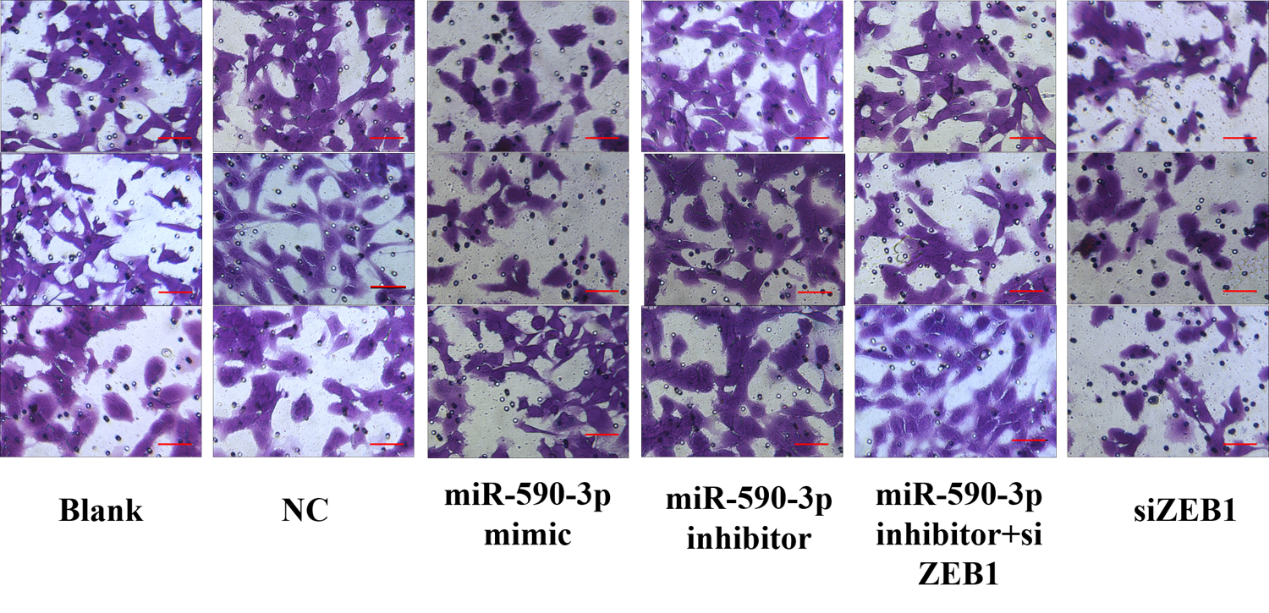


**Figure S4. Effect of interfering with the expression of ZEB1 on migration of Human Cardiac Fibroblasts (200**$\boldsymbol{\times}$**)**. Bars: 50 μm. NC: negative control.
